# Using SED-ML for reproducible curation: Verifying BioModels across multiple simulation engines

**DOI:** 10.1101/2025.01.16.633337

**Published:** 2025-01-20

**Authors:** Lucian Smith, Rahuman S. Malik-Sheriff, Tung V. N. Nguyen, Henning Hermjakob, Jonathan Karr, Bilal Shaikh, Logan Drescher, Ion I. Moraru, James C. Schaff, Eran Agmon, Alexander A. Patrie, Michael L. Blinov, Joseph L. Hellerstein, Elebeoba E. May, David P. Nickerson, John H. Gennari, Herbert M. Sauro

**Affiliations:** 1 Department of Bioengineering, University of Washington, Seattle, WA, USA; 2 European Molecular Biology Laboratory, European Bioinformatics Institute (EMBL-EBI), Cambridge, UK; 3 Icahn School of Medicine at Mount Sinai, New York, NY, USA; 4 University of Connecticut School of Medicine, Farmington, CT, USA; 5 Department of Biomedical Informatics and Medical Education, University of Washington, Seattle, WA, USA; 6 eScience Institute, University of Washington, Seattle, WA, USA; 7 Department of Medical Microbiology and Wisconsin Institute of Discovery, University of Wisconsin-Madison, Madison, USA; 8 Auckland Bioengineering Institute, University of Auckland, Auckland, New Zealand

## Abstract

The BioModels Repository contains over 1000 manually curated mechanistic models drawn from published literature, most of which are encoded in the Systems Biology Markup Language (SBML). This community-based standard formally specifies each model, but does not describe the computational experimental conditions to run a simulation. Therefore, it can be challenging to reproduce any given figure or result from a publication with an SBML model alone. The Simulation Experiment Description Markup Language (SED-ML) provides a solution: a standard way to specify exactly how to run a specific experiment that corresponds to a specific figure or result. BioModels was established years before SED-ML, and both systems evolved over time, both in content and acceptance. Hence, only about half of the entries in BioModels contained SED-ML files, and these files reflected the version of SED-ML that was available at the time. Additionally, almost all of these SED-ML files had at least one minor mistake that made them invalid. To make these models and their results more reproducible, we report here on our work updating, correcting and providing new SED-ML files for 1055 curated mechanistic models in BioModels. In addition, because SED-ML is implementation-independent, it can be used for *verification*, demonstrating that results hold across multiple simulation engines. Here, we use a wrapper architecture for interpreting SED-ML, and report verification results across five different ODE-based biosimulation engines. Our work with SED-ML and the BioModels collection aims to improve the utility of these models by making them more reproducible and credible.

## Author summary

Reproducing computationally-derived scientific results seems like it should be straightforward, but is often elusive. Code is lost, file formats change, and knowledge of what was done is only partially recorded and/or forgotten. Model repositories such as BioModels address this failing in the Systems Biology domain by encoding models in a standard format that can reproduce a figure from the paper from which it was drawn. Here, we delved into the BioModels repository to ensure that every curated model additionally contained instructions on what to do with that model, and then tested those instructions on a variety of simulation platforms. Not only did this improve the BioModels repository itself, but also improved the infrastructure necessary to run these validation comparisons in the future.

## Introduction

Model reproducibility is a broad concern of the entire scientific community [1]; in general, reproducibility is the first step for building upon prior scientific work. In a biosimulation publication, reproducibility means that the scientific claims of the paper can be verified by reproducing the execution of the computational model. Results from computational models are in theory easily reproducible, as the mathematics involved are well-understood, and the calculations should be the same regardless of the particular software or operating system used. However, modeling has been no more immune to reproducibility failures than any other branch of science [2]. The BioModels Database [3] was established in 2005 to combat this problem, enlisting the help of curators to re-encode biological models from published papers into the System Biology Markup Language (SBML [4]), and then carrying out an experiment to verify some results from the publication. BioModels is a database of mathematical models of biomedical systems that has grown to include 1073 curated models (as of June 2024) collected from peer reviewed articles that have been manually curated to ensure they reproduce published results. The curation work has been carried out by the European Bioinformatics Institute (EMBL-EBI), which provided a consistent protocol for how curation is carried out.

The process of curating a model in the BioModels collection begins with encoding the model into a standard format, typically the Systems Biology Markup Language (SBML). Models are published in various representations, including being described as equations in the text, so standardization is essential. Next, the curator selects a “results” figure from the publication and attempts to reproduce this figure using a biosimulation engine. For example, a model described using ordinary differential equations (ODEs) might be simulated using COPASI [5] or Tellurium [6]. The curator will then provide the output figure from the biosimulation engine, and a brief description of the curation work (E.g., *“We reproduced* figure 2a, *using COPASI v3.5. Note that the units in this figure differ from the publication. The correct value of the parameter K is 0.1 and the value reported in the manuscript is incorrect”*). Finally, model elements are annotated, following an established procedure for annotation based on MIRIAM guidelines [7] (the Minimal Information Requested In the Annotation of biochemical Models). Once the curation was complete, all other required and auxiliary files are placed into an archive for that entry.

Although this process establishes that a set of results can be reproduced, there are some shortcomings. Curation often involves some guessing on the part of the curator, which can make the process non-repeatable if those choices are not recorded. The curation results may depend on which figure is being reproduced, simulator settings, time steps, initial conditions, and other considerations that must be inferred from the published article. Recognizing these challenges, the Simulation Experiment Description Markup Language (SED-ML) [8] was developed in 2011 to store this information in a reproducible format. However, adoption of this standard has been slow. Although five SED-ML files were created by BioModels curators by hand, it wasn’t until COPASI added SED-ML support in 2014 that it became possible to create SED-ML consistently, and since 2017, BioModels curators have used this functionality to produce SED-ML for over 380 BioModels entries.

The need for SED-ML emerged as the modeling community realized that “what you do with a model” was just as important to standardize as the modeling language itself. SED-ML aims to capture the process of loading a model, potentially changing model values, running a simulation experiment such as a timecourse simulation or parameter scan, and collecting the results as tables of data (reports) or plots. Importantly, none of these steps are connected to any particular biosimulation engine, and thus, SED-ML provides a language for simulation that can be applied to almost any biosimulator.

For this work, the Center for Reproducible Biomedical Modeling (https://reproduciblebiomodels.org/), has collaborated with the BioModels team at EMBL-EBI (https://www.ebi.ac.uk/biomodels/) to improve the completeness and quality of the curation of the BioModels collection. To address all of these problems, we have taken a systematic approach to leveraging SED-ML. For BioModels, curation typically means reproducing results with a single biosimulator different from the one used by the modeller. With SED-ML, we now have the potential of *verification* of results across multiple engines. This enhances the possibility of reusing and extending published results, both because researchers may prefer particular engines and because results verified across multiple biosimulators are more credible.

As we describe, much of our work has been to produce valid and functional SED-ML files for all of the curated BioModels so that researchers can independently verify that a model can be executed on some simulation engine. SED-ML has been expanded over the years, and can now describe a wide range of computational experiments (e.g., parameter scans, and analysis of qualitative models) [9]. We validated and updated the existing SED-ML files, and we used the COPASI SED-ML export feature to produce additional SED-ML when COPASI files were available.

Unfortunately, for about half of the curated entries, no computer-readable record of how to replicate the published figure was saved. In these cases, the only information remaining is the very brief “curator comments” and an image file of the generated figure. As a solution, we have created a “template” SED-ML file for these models, because even though the template SED-ML will not reproduce any publication figure, it can still be used to determine if the model will behave the same when used in different biosimulation engines, and can be used as a springboard to produce SED-ML which fully recapitulates published results [10].

Of the 476 models with either an existing SED-ML file or a COPASI file, we were able to get 386 models (81%) to run on multiple biosimulation engines and produce the same results. Of the remaining 579 models with only ‘template’ SED-ML, we were able to get 546 (94%) to run on multiple biosimulation engines and produce the same results. Although these early results do raise some concerns about the ability to reproduce published results across different engines, the use of SED-ML provides a way forward. These formal specifications can now be used to investigate the differences discovered across biosimulation engines, verifying not only the models themselves but the simulation engines as well.

## Methods

**Fig 1.**
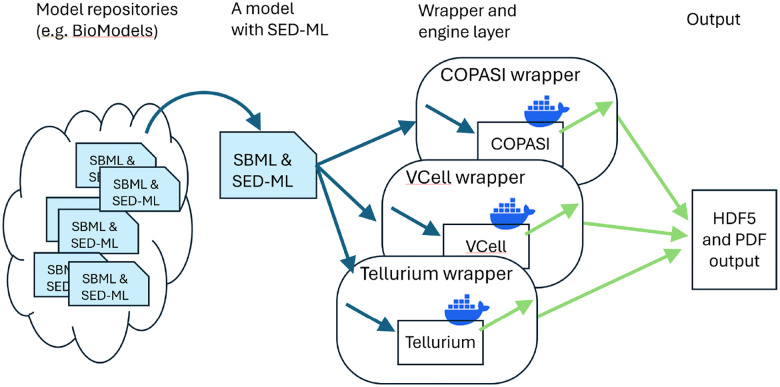
SED-ML and wrappers that allow researchers to replicate results across multiple simulators. See Shaikh et al. [11] for more details about the biosimulation engine wrapper architecture.

Although there are many ways to improve reproducibility of systems biology modeling, here we focus on the ability of researchers to replicate published simulation results and then to verify them using different biosimulation engines. As Figure 1 shows, as a first step, model repositories such as BioModels should include SED-ML to describe the steps needed to replicate the results. We have created a wrapper architecture at BioSimulators.org [11] where this information can then be interpreted and sent to different biosimulation engines. The majority of models in the curated BioModels collection are ODE models (1055 of 1073); therefore, we have selected five well-tested biosimulation engines, and built and tested wrappers for these: COPASI [5], Tellurium [6], VCell [12], (shown in Fig. 1), PySCeS [13] and Amici [14]. As we describe, many curated models (but not all) could indeed be verified across more than one of these biosimulation engines.

### SED-ML Wrapper Development

Most biosimulation software engines are developed independently, each with its own unique interfaces. While many offer APIs (Application Programming Interfaces) for accessing their capabilities, these APIs differ from one engine to another, requiring customized handling of input and output data to match each engine’s specific API. Our solution, shown in Figure 1, is a “wrapper” architecture: each wrapper translates SED-ML instructions to API calls for the respective biosimulation engine, all within Docker containers to ensure platform independence and stability over time. By using this approach, SED-ML serves as a *lingua franca*, enabling the same procedure to be executed across different wrapped biosimulation engines. This allows researchers to precisely verify results using standardized inputs and outputs across multiple engines.

To carry out this work, we took a snapshot in time from June of 2024 of the curated branch of BioModels, working with a copy of the entire curated branch of 1073 models, 1055 of which were ODE models. Each of these entries is stored as an archive that includes the SBML file of the model, as well as all ancillary files associated with that model.

These archives are stored in Open Model EXchange (OMEX) files, a format standardized by the biomodeling community as a standard way of collecting model experiment files together [15]. This format is essentially a ZIP file of the collected files, plus a manifest file listing and describing each file. In 2017, the BioModels database was updated to allow each entry’s collection of files to be downloaded as an OMEX file, with the manifest file noting which file was the canonical model file for that entry. In some cases, separate OMEX files were also included as auxiliary files themselves.

Each wrapper takes an OMEX file (containing at least one SED-ML and one SBML file) as input, translates the SED-ML instructions into simulator-specific commands, collects the output of any simulations from those simulators, and exports all outputs. Tabular data is exported as HDF5 files [16] (a format similar to CSV, but allowing multi-dimensional data and annotations), and figures are exported as PDF files. While non-semantic differences in figure output prevent the automatic comparison of figure data, the numbers inside the HDF5 files can be compared directly. In this way, we can verify whether the SED-ML files will produce the same output on multiple biosimulation engines.

In the process of developing the wrappers, we discovered a number of SED-ML constructs that were ill-defined in the specification (which at that time was Level 1 Version 3, released October 2017 [17]). We coordinated with the SED-ML community and the SED-ML Editors in particular to clarify and update the specification. As a result, the SED-ML Level 1 Version 4 specification was released in September 2021 [18] (with some members of our team joining as SED-ML editors), which included over 100 changes due to issues discovered during this process. To our knowledge, development and curation using our SED-ML wrappers is the first systematic exploration of different simulators’ interpretations of SED-ML files.

### BioModels retro-curation and updates

At EMBL-EBI, the BioModels curation process (described above) has generally been focused on the SBML model file, with all ancillary files treated as secondary to the SBML file. These secondary files had never before been systematically checked for accuracy, and our first goal for this project was to ensure that the files were at least valid. We created a series of Python scripts to perform this task, validating all 24 file types found across the entire database, from SED-ML files to original COPASI files to images and PDF documents. Occasional errors were found (and corrected) in almost every category, from incorrect character sets to zero-sized files to invalid characters in filenames, and a host of other problems. As one example, we found some auto-generated PDF documents which contained only an error message from the converter used to create the file. Invalid files were either corrected or removed.

We also validated the final annotation step of curation by ensuring that every annotation URL in the SBML file pointed to an actual entry in an existing ontology. A variety of errors were caught by this check, including simple typos of the name of the ontology, or leaving out parts of the required format for the reference. Incorrect entries were manually corrected, consulting the original paper for references when necessary.

We then focused on SED-ML files. In a perfect world, we would be able to ensure that each entry contained a SED-ML file with instructions for how to reproduce a figure from the curated paper. This was unfortunately impossible to accomplish in an automated way, as no good algorithm exists to automatically numerically compare two images. However, a number of BioModels entries contained either SED-ML files exported from COPASI, or the original COPASI files themselves. Even though the SED-ML files were usually invalid, they could still be fixed, and COPASI itself could be used to generate (equally invalid, and equally fixable) SED-ML files from the original COPASI files. In most cases, the COPASI file would have been created when the curator was reproducing a figure from the paper, and thus its exported SED-ML file would also contain the instructions necessary to reproduce that same figure. For entries without SED-ML or COPASI files, we created ‘template’ SED-ML files that would at least demonstrate that a straightforward simulation of the model could be replicated across multiple biosimulation engines, even if those simulations were not what was used in the original publication.

SED-ML files exported from COPASI are always invalid in the BioModels context, because COPASI will always generate SED-ML that points to an SBML file named ‘model.xml’, which is not the correct name. This is true both for the SED-ML files we generated from the original COPASI files as well as the existing legacy SED-ML files found in existing BioModels entries, making all of them invalid apart from the five that had been created by hand. However, this problem is trivial to fix programmatically whenever only one SBML file is present, or the correct file could be deduced from other clues when multiple SBML files were present. In this way, we could fix existing SED-ML files or export new ones when an original COPASI file existed for the entry.

Helpfully, the current version of COPASI (4.43) is backwards compatible with older COPASI files, meaning that we could create SED-ML for many entries that had been created before SED-ML even existed. When both a COPASI model file and a previously-exported SED-ML file were present for the same model, we hand-compared the the existing file to one recreated from the COPASI file. In most cases, the files were nearly identical or the newly recreated files were more precise and detailed. In three cases, the existing SED-ML file had more details than the re-generated version. We hypothesized this was due to the SED-ML having been exported from a working COPASI curation session which was not subsequently stored as a COPASI file.

Even so, ‘pointing to an incorrect model’ was far from the only problem present in the files: references were found to nonexistent model elements; simulations were defined and never used; duplicate elements were present; pointers to model elements were incorrectly formatted; simulation parameters were incorrectly applied; and many other issues were discovered. Most of these fixes had to be applied on a per-entry basis, with problems uncovered by testing and individual fixes applied.

Most of these problems were ferreted out and fixed by using our wrappers to run the SED-ML on biosimulations.org, or by using Tellurium’s native SED-ML interpreter. Sometimes the problems would be obvious, because the simulator would fail with a clear error message–incorrect element references fell in this category more often than not. More issues were discovered when comparing two different simulators to ensure they both produced the same results. This enabled us to discover and fix/report bugs all along the chain: in COPASI’s SED-ML exporting routine, in the SED-ML wrappers, in the SED-ML interpretation library, and in Tellurium’s native SED-ML interpreter.

Another benefit of this project was that these newly-created SED-ML files essentially formed a new test suite of real-world SED-ML examples. They uncovered a wide variety of issues in our wrappers, which we were then able to fix, and also served as test examples for COPASI and Tellurium’s native SED-ML interpreters. In the end, the wrappers and the native interpreters now all cover a much wider range of SED-ML inputs than they did before, and have fewer problems.

For the 579 entries with no SED-ML nor a COPASI file that could be used to generate SED-ML, we created a ‘template’ SED-ML file, encoding a simple time course experiment into it. The model is loaded, a timecourse simulation is performed for ten time units, and finally, the levels of all variable species are exported, both as a table of values per time point, and as a plot. Of course, these files do not match any figures from the original publication, but can still be used for verification purposes: if two simulators produce the same output from this template SED-ML, it shows that the model itself is robust to interpretation. It is also much easier to edit an existing SED-ML file than to create one from scratch, making future efforts to reproduce published figures simpler [10].

We created a GitHub repository (https://github.com/sys-bio/temp-biomodels) with a copy of the curated branch of the BioModels database. Python scripts were added that could process each BioModels entry, analyzing and validating every file, and producing a new entry with only valid files. These new files were then pushed to the official biomodels repository at http://biomodels.net, where they all show up as the latest versions of the curated models. This means that executable SED-ML is now available for all of these models, each noting whether it is a ‘template’ file or one drawn from the curation process.

Moving forward, the scripts developed can be used by EMBL-EBI curators to perform more robust curation: not only can the files they collect be validated, but they can now additionally store validated SED-ML files and test them across multiple biosimulation engines on biosimulations.org.

## Results and Discussion

All 1073 curated entries retrieved from the BioModels database have been updated, with every file now validated or replaced. All 1055 ODE entries have a valid SED-ML file that can be used to simulate the model in some capacity: there are 579 “template” SED-ML files, and 476 SED-ML files that replicate a figure.

**Fig 2.**
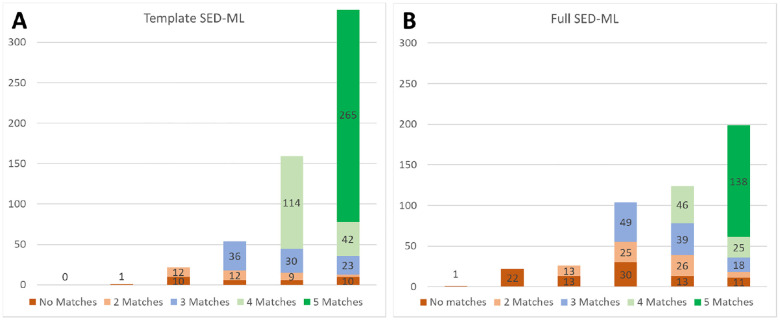
Template (A) and Full SED-ML (B) replication across five wrapped simulators.

As shown in Table 1, the great majority of these files run with the Tellurium and Copasi wrappers, and hundreds of models run successfully using our other three SBML wrappers. Tellurium has the most, simply because we focused our efforts on the wrapper for this platform since we have the most knowledge and control over this biosimulation engine. Work is ongoing to update the other wrappers to match. Figure 2 shows the distribution of successful runs and replications for both template and “full” SED-ML; i.e., SED-ML code for the 470 models that actually aim to reproduce a specific figure.

For all models with successful runs on at least two wrapped simulators, we defined a successful replication as having results that matched to a relative tolerance of 0.0001 and absolute tolerance scaled by the range of output values for that variable. As can be seen, of the ‘full’ SED-ML file entries, one did not run on any of the five simulator wrappers, and 22 only ran on one. An additional one ‘template’ SED-ML file entry only ran on a single simulator. These entries were unable to be replicated because two sets of results did not exist to be compared. Of the remaining entries with the relatively straightforward template SED-ML, replication was possible in 578 cases, and successfully verified in 546/578 cases (94%). For the potentially more complicated entries with full SED-ML, 453 cases could be replicated, with verification shown for 386/453 cases (85%).

Initially, our results for replicability across engines were much weaker. Specifically, an earlier draft of this manuscript showed much lower success rates for simulation engines other than Tellurium. However, once these early results were circulated among the community, a broad and significant effort was invested by many institutions and research groups. Bugs were fixed and improvements were made to the SED-ML files themselves (such as fixing references or ranges), the biosimulation wrappers (such as fixing SED-ML interpretation, and improving edge case handling), the infrastructure that runs the wrappers (such as improving latency and robustness), and the simulation engines themselves (such as improving SBML interpretation and handling of simulation edge cases). Thus, an important contribution of our work has been to stimulate this type of community-based work to improve the consistency and accessibility across a range of simulation engines. This work is not finished, and is expected to continue into the future, spurring further improvements.

All of these improvements are freely available: the SED-ML files are now available at part of the BioModels entries at http://biomodels.net, the wrappers are available on github at https://github.com/orgs/biosimulators/repositories, the Docker images are available at https://biosimulators.org/simulators, the wrappers can be run online at https://biosimulations.org/runs/new, results from verified BioModels entries (and others) can be viewed and downloaded from https://biosimulations.org/projects, and the improved simulators can all be accessed from their authors’ web sites.

Furthermore, additional improvements in consistency will now be easier to carry out. Simulators that fail to produce results from particular BioModels entries now have a test harness where improvements to the simulator or simulator wrapper can be checked as they are expanded to handle a wider variety of inputs. Cases where a simulator’s results for a given BioModel entry did not match replicated results from two or more other simulators can now be pulled out and examined: at least 30 such models exist for every single simulator in our study.

It is now also increasingly possible to usefully critique the models themselves: how reproducible are they? How sensitive are the published results to the parameter values? Can they be extended? With a system in place that makes re-simulation straightforward, these questions become much easier to answer.

For both parts of Figure 2, the height of the full bar indicates the number of models that successfully ran on zero (left) through five (right) of our wrapped simulators. Both the leftmost ‘zero’ and ‘one’ columns could only have no matches, as there were not two successful simulations to compare against each other. For the most part, the ‘full’ SED-ML entries did not run on as many simulators as the ‘template’ SED-ML entries, as the latter use a wider variety of SED-ML features, not all of which are supported by all the wrappers or simulators.

Recall that this study stops short of ensuring that the simulator runs actually reproduce a figure from the original publication; for many models, all we have is brief “curator comments”, so this process cannot be easily automated. We expect that most of the 476 entries (see Figure 2b) that contained SED-ML or COPASI files will indeed match a figure from the paper, as those files were used during curation to reproduce a figure. However, as the inclusion of a COPASI file was not a formal part of the original curation process, this is not guaranteed, so every model will need to be checked by hand. The entries with template SED-ML will not match published results, and the SED-ML would need to be modified to match the original computational experiment.

## Conclusions and future directions

This project demonstrated that even manually curated models can be challenging to reproduce without appropriate specifications for how to carry out the experiment. We demonstrate the utility of the SED-ML language for capturing these specifications by comprehensively creating or improving valid SED-ML files for 1055 ODE-based models in the BioModels database, all but one of which could be successfully run in at least one of our five wrapped simulation engines. For 88% of the models (928/1055), we were able to show that two separate simulators produced the same results within reasonable tolerances. Work still needs to be done to fully replicate the original curator’s work in reproducing the published results, as there is no formal record of how these models were originally curated. We have begun some of this work, and early results are encouraging [10].

Moving forward, this project demonstrates the need for improved SED-ML generation tools. Model-building environments should be able to generate SED-ML for any given experiment or execution of a model. The COPASI tool comes close to this, but it only creates SED-ML for a single model/experiment/plot combination, which isn’t sufficient for experiments involving multiple models, nor plots involving multiple experiments. In addition, it cannot export model changes (i.e. ‘in this figure, the value of n was 2, and the value of S1 was 2.42’). As an alternative approach, the Tellurium Python environment includes the phraSED-ML library, which makes it easy to create and execute a SED-ML file, but ‘creating a SED-ML file’ has to be a goal of the user, who otherwise will just use Python directly to carry out their experiments.

In general, auto-generating SED-ML will be challenging. In a GUI environment like COPASI, model changes are executed and not stored, so an experimental protocol that includes ‘change this value’ will not encode that change into the SED-ML, but will instead export a different initial model with those new values. In a scripting environment like Tellurium, common scripting behaviors like loops will commonly be encoded into the scripting environment itself, making it difficult to then export to SED-ML. Any tool that produces SED-ML but requires the user to encode these procedures separately from the user’s usual workflow must therefore offer significant benefits in exchange.

Finally, an important contribution of our work is to demonstrate the value of the wrapper architecture for biosimulation engines, and how that can support model verification. As we have shown, although many models do produce the same results across multiple engines, some models do not execute on some engines, and some models produce different results across engines. All of our scripts and wrappers are available via GitHub (https://github.com/biosimulators/), and we hope that model builders will be interested in using these to test their own experiments across multiple biosimulators. In the future, we plan to build a REST API for verifying any model coded in SBML (and/or OMEX archives with SBML) across multiple biosimulation engines.

Although the BioModels database has carried out ground-breaking work developing a curated model repository, until now, the database did not include enough information to fully replicate published results. Here, we have made significant inroads towards solving this problem, by providing valid SED-ML files for all models, as well as providing SED-ML files that can be used to reproduce published results in almost half of the collection. Moving forward, additional work on improved wrappers is needed, and model development environments should be designed to make it easier to auto-generate SED-ML. Without strong tool support and systematic checking of results, it is too easy for errors or omissions to accumulate, which ultimately makes published results non-reproducible. To ensure reproducible results, we strongly encourage authors to submit their models, accompanied by correct SED-ML files, to BioModels or comparable resources as part of the publication process. With the use of SED-ML, we have demonstrated the ability to *verify* published results across multiple biosimulation engines.

## Figures and Tables

**Table 1.** Successful runs for each simulator of the 1055 currently curated ODE-based BioModels.

Simulator	Successful runs
Tellurium wrapper	1036
Copasi (basico) wrapper	1032
VCell wrapper	893
Amici wrapper	758
PySCeS wrapper	711
At least two of the above	1031
